# Thomas A. Long

**Published:** 1901-12-15

**Authors:** 


### THOMAS A. LONG.

Thomas A. Long, the well known and greatly esteemed traveling man for the S. S. White Dental Manufacturing Company, is dead. This will be a shock to all who knew him, and there are few who do not. He was a most genial and companionable gentleman, and was known as one of the famous "story tellers." He had a full repertoire of amusing and instructive anecdotes and few people excelled him in their narration. His business was selling dentists' supplies, largely of late years to the dealers only. In his long acquaintance with dentists and in endeavoring to supply their needs he became an expert, and was always welcomed to the best and busiest offices in the land, because he transacted his business in a gentlemanly manner and had tact enough to know when he was through. He never sought to sell his wares to any one he knew did not need or want them. He is one of the men we shall all miss and mourn. We are, however, thankful to have known him, and for the cheerful and hopeful view of life which he always manifested. May the tribe of such as he was increase. Mr. Long died at his home in Oxford, Md., Nov. 5, 1901, of apoplexy.

